# Automated Local Measurement of Wall Shear Stress with AI-Assisted Oil Film Interferometry

**DOI:** 10.3390/s26020701

**Published:** 2026-01-21

**Authors:** Mohammad Mehdizadeh Youshanlouei, Lorenzo Lazzarini, Alessandro Talamelli, Gabriele Bellani, Massimiliano Rossi

**Affiliations:** 1 Department of Industrial Engineering, University of Bologna, Via Montaspro 97, 47121 Forlì, Italy; mohammad.mehdizadeh2@unibo.it (M.M.Y.); alessandro.talamelli@unibo.it (A.T.); massimiliano.rossi13@unibo.it (M.R.); 2 Interdepartmental Centre for Industrial Research in Aerospace, University of Bologna, Via Baldassarre Carnaccini, 12, 47121 Forlì, Italy; lorenzo.lazzarini7@unibo.it

**Keywords:** wall shear stress, optical sensing, Oil-Film Interferometry, deep learning, YOLO, VGG16

## Abstract

Accurate measurement of wall shear stress (WSS) is essential for both fundamental and applied fluid dynamics, where it governs boundary-layer behavior, drag generation, and the performance of flow-control systems. Yet, existing WSS sensing methods remain limited by low spatial resolution, complex instrumentation, or the need for user-dependent calibration. This work introduces a method based on artificial intelligence (AI) and Oil-Film Interferometry, referred to as AI-OFI, that transforms a classical optical technique into an automated and sensor-like platform for local WSS detection. The method combines the non-intrusive precision of Oil-Film Interferometry with modern deep-learning tools to achieve fast and fully autonomous data interpretation. Interference patterns generated by a thinning oil film are first segmented in real time using a YOLO-based object detection network and subsequently analyzed through a modified VGG16 regression model to estimate the local film thickness and the corresponding WSS. A smart interrogation-window selection algorithm, based on 2D Fourier analysis, ensures robust fringe detection under varying illumination and oil distribution conditions. The AI-OFI system was validated in the high-Reynolds-number Long Pipe Facility at the Centre for International Cooperation in Long Pipe Experiments (CICLoPE), showing excellent agreement with reference pressure-drop measurements and conventional OFI, with an average deviation below 5%. The proposed framework enables reliable, real-time, and operator-independent wall shear stress sensing, representing a significant step toward next-generation optical sensors for aerodynamic and industrial flow applications.

## 1. Introduction

Wall shear stress (WSS), denoted as $\tau_{w}$, is a fundamental quantity in aerodynamics and fluid mechanics, directly linking the surface stress distribution to momentum transfer and drag generation. Its accurate determination is critical not only for fundamental studies of wall-bounded turbulence and model validation [1,2,3,4], but also for applied domains such as flow control, aerodynamic optimization, and drag-reduction technologies [4]. Despite decades of development, reliable local measurement of WSS in real time remains an experimental challenge.

Traditional techniques for wall shear stress characterization are generally classified into indirect and direct measurement methods. Indirect approaches, such as pressure-drop measurements or velocity-gradient determination via hot-wire anemometry or PIV, infer wall stress from flow properties measured away from the wall. Pressure-drop methods deliver accurate global averages but lack spatial resolution and require fully developed pipe flow conditions [5,6]. Similarly, velocity-gradient techniques enable spatial mapping but demand complex and costly setups along with detailed assumptions about the flow field [7]. Conversely, direct methods locally capture the interaction between flow and wall. MEMS and floating-element shear sensors provide direct, local sensitivity, but are typically intrusive, require meticulous calibration, and are susceptible to mechanical disturbances or vibrations [8,9]. Optical techniques, especially Oil-Film Interferometry (OFI), are part of the direct measurement group, offering a straightforward and well-established optical link between oil film thinning rate and local wall shear stress [8,9,10,11]. The OFI technique enables quantitative, spatially resolved, and non-invasive WSS estimation using simple implementations, extensively validated in the literature [12,13,14]. Nevertheless, conventional OFI remains strongly operator-dependent and ill-suited for automated or real-time measurements: droplet irregularities, lighting variations, and subjective selection of interrogation windows introduce significant uncertainty, limiting practical sensor applicability.

The recent progress in artificial intelligence (AI) and computer vision offers a powerful opportunity to overcome these limitations. Deep convolutional neural networks (CNNs) have demonstrated outstanding capabilities in complex image-processing tasks across scientific fields, including turbulence analysis [15,16,17,18,19,20,21,22,23], thermal transport [24,25], and experimental fluid mechanics [26,27,28,29,30,31,32,33]. Taking advantage of these developments, this work introduces an AI-assisted OFI (AI-OFI) framework that converts a classical optical visualization method into a self-calibrating, real-time wall shear stress sensor.

The proposed method integrates two convolutional architectures—YOLO-v8 [34] for droplet detection and VGG16 [35] for fringe-based regression—combined with a smart interrogation-window algorithm based on the 2D Discrete Fourier Transform. The hybrid system is trained and validated using extensive experimental data from the CICLoPE long-pipe facility [36], enabling the first demonstration of fully automatic quantitative WSS sensing in turbulent pipe flow in a broad range of Reynolds numbers.

The remainder of the paper is structured as follows: Section 2 reviews the theoretical background of Oil-Film Interferometry and deep-learning-based image analysis. Section 3 presents the experimental setup and AI-OFI methodology, Section 4 discusses the results and validation, and Section 5 summarizes the main findings and future developments for real-time optical shear sensors.

## 2. Background

### 2.1. Theory of Oil Film Interferometry

**Figure 1:**
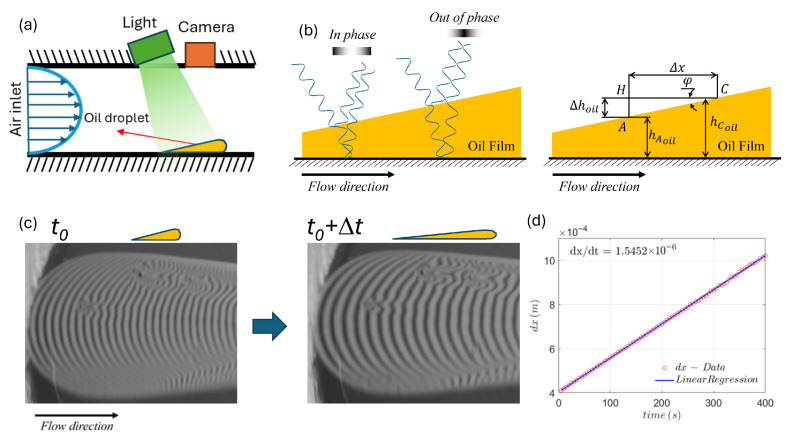
(**a**) Schematic of oil droplets in the long pipe influenced by air flow and output images. (**b**) Measurement principle based on interference fringes produced by Fizeau Interferometry: the light source produces constructive/destructive interference depending on the film thickness. (**c**) Snapshots of the OFI with Fizeau fringes at different instants of time. (**d**) Sample OFI regression of the presented data.

The sensing element in the OFI technique is a thin oil droplet that is deposited on a surface exposed to the flow [8]. As the fluid passes over the considered surface, the oil thins as a consequence of the WSS exerted on the oil–air interface (Figure 1a). The thickness of the oil film can be measured due to the phenomenon of amplitude splitting, or Fizeau interferometry, as shown in the schematics of Figure 1b,c. Consider a source of monochromatic light of wavelength λ that strikes the film at an angle α. Part of the light is reflected from the oil/air surface, whereas another part is refracted into the film. As it reaches the solid surface, the refracted light is reflected away and travels back through the oil film [9]. When focused by a lens, these two beams are combined and interfere constructively or destructively depending on their phase difference. Assuming that the slope of the oil interface ϕ is very small, the difference in height between two bright (or dark) peaks $\Delta h_{\mathrm{oil}}$ depends solely on λ, α, and the refractive index of oil, $n_{oil}$, and air, $n_{air}$,

(1) $$\Delta h_{\mathrm{oil}}=\frac{\lambda_{L}}{2\cdot \sqrt{n_{\mathrm{oil}}^{2}-n_{\mathrm{air}}^{2}\cdot \sin^{2}\left(\alpha \right)}}.$$

Under the assumption that the dominant contribution to the movement of the thin film is caused only by tangential stress at the wall in one direction, we can describe the height of the oil film $h_{\mathrm{oil}}$, taking advantage of the equation of the thin oil film.

(2) $$\frac{\partial h_{oil}}{\partial t}+\frac{\partial }{\partial x}\left(\frac{\tau_{w}h_{oil}^{2}}{2\mu_{oil}}\right)=0;$$

Under the further assumption of constant $\tau_{w}$ at the wall, we can find an analytical solution for Equation (2) and the corresponding slope of the droplet interface [9].

(3) $$h_{oil}=\frac{\mu_{oil}}{\tau_{w}}\frac{x}{t}\Rightarrow \frac{\partial h_{oil}}{\partial x}=\frac{\mu_{oil}}{\tau_{w}}\frac{1}{t}.$$

The slope is linear and inversely proportional to time, so that $\frac{\partial h_{oil}}{\partial x}\approx \frac{\Delta h_{oil}}{\Delta x}$, where Δx corresponds to the distance between two bright peaks, as shown in Figure 1c. Rearranging and calculating over time, remembering that all quantities are constant over time except Δx, it is easy to show that

(4) $$\tau_{w}=\frac{\mu_{\mathrm{oil}}}{\Delta h_{\mathrm{oil}}}\frac{\partial }{\partial t}\Delta x.$$

Since $\mu_{\mathrm{oil}}$ and $\Delta h_{\mathrm{oil}}$ are constants determined by the material properties of the oil and by the experimental settings, we can determine the WSS from a measurement of the rate of change in the distance between fringes Δx. From an experimental point of view, the term $\left(\frac{\partial }{\partial t}\Delta x\right)$ is calculated from a series of (n) photographs of a droplet taken at equal time intervals. An example of photographs taken at two different time instants is shown in Figure 1c. An image processing procedure is then used to extract the average distance of the fringes Δx as a function of time. The wall shear stress, directly proportional to $\left(\frac{\partial }{\partial t}\Delta x\right)$, can be readily extracted from a linear interpolation of the data (Figure 1d).

While this approach is conceptually straightforward, a major practical challenge is given by the selection of the interrogation window (IW) for the calculation of the fringe distance Δx. The actual deformation of the oil droplet is more complex than the planar geometry assumed in the theory, so that the value of Δx can be strongly affected by the position of the IW. Furthermore, the overall position of the droplet can shift slightly during the experiment due, for instance, to vibrations of the setup. Finding an algorithm able to consistently identify the best IW is very challenging; therefore, conventional OFI measurement typically relies on a user intervention for the selection of the IW [6,11]. This approach, however, presents two significant shortcomings: it strongly relies on the expertise of the user and is not suitable for real-time, continuous measurements.

### 2.2. Deep Learning for Image Analysis

Deep learning is a powerful AI tool based on deep neural networks with multiple hierarchical layers of feature extraction, widely implemented in different scientific research fields. In the case of image analysis, convolutional neural networks (CNN) are used. In the fluid dynamics field, deep learning is extensively used in both experimental [22] and numerical [16] studies. In turbulence flow studies, due to the very time-consuming CFD approaches, deep learning for reducing calculation time received a lot of attention [15,17,18,19,20,21,23], also in turbulent heat transfer [24,25]. Moreover, in complex flows, deep learning showed potential for improvements in simulation and prediction of flow field [26,27,28]. Particle image velocimetry is another related method that exhibits good compatibility with deep learning techniques due to the required image processing steps [29,30,37]. In particular, in the field of microfluidics, deep learning has been used to improve the accuracy of particle tracking techniques based on defocusing [31,32,38], enabling new capabilities such as the determination of three-dimensional orientation of non-spherical particles and micro-organisms [33].

## 3. Materials and Methods

### 3.1. Experimental Setup

**Figure 2:**
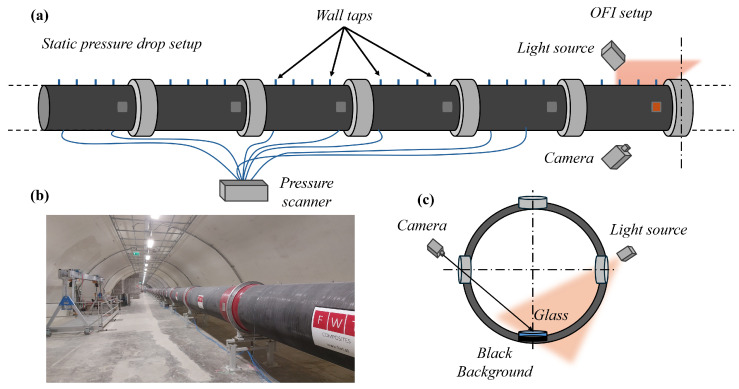
(**a**) Experimental setup for Wall shear stress measurements using both OFI and static pressure drop, (**b**) Image of the Long Pipe Facility, (**c**) schematic view of the OFI acquisition setup.

The experiments were carried out in the long pipe of the Centre for International Cooperation in Long-Pipe Experiments (CICLoPE). The closed-loop circular pipe is 111.5 m long with a radius of 0.4505 m, resulting in an L/D of 124 [36]. A picture of the long pipe facility is shown in Figure 2b. The experiments were performed in the test section located at 110 m = 244R downstream with respect to the inlet, thus considering the flow fully developed. The pipe is constructed from twenty-two 5 m long carbon fiber sections plus a final 1.5 m long section, all produced using filament-winding technology. This method achieves a surface roughness of $k_{rms}$ < 0.2 μm $\left(k^{+}<0.02\right)$, thus ensuring smooth-wall conditions. Each section is fitted with four axially spaced static pressure taps and four radially distributed access ports of 150 mm in diameter, providing access to the pipe and the possibility to monitor pressure drop across the entire pipe length. The friction Reynolds number was in the range $4794≲Re_{\tau }≲47,015$, with a centerline velocity range of 3.837 m/s ≤ $U_{cl}$ ≤ 48.60 m/s.

The pressure drop across the pipe was acquired for each experiment using wall pressure taps integrated into the pipe walls, with a hole dimension of 2 mm and placed on the top of the pipe wall at a distance of approximately 1 m from each other, as shown in Figure 2a. The static pressure was acquired with a DTC Initium multi-pressure transducer (Danatech srl, Milan, Italy) with a range of 10 Torr (1.33 kPa) and a resolution of ±0.05% full scale. The acquisition frequency was set to 100 Hz, while the acquisition time was synchronized with the OFI experiments. Ambient conditions were monitored using an absolute MKS Baratron transducer (MKS, Andover, MA, USA) having a range of 1000 Torr (133 kPa). The temperature was monitored using PT100 sensors placed in two different positions of the test section of the pipe: at the wall and in the center-line.

### 3.2. Conventional OFI Measurements

The experimental setup for the reference OFI measurement is presented in Figure 2c. A horizontal glass surface [39] having a refraction index equal to 1.5 was placed on the bottom wall of the test section. For each experiment, three oil droplets were carefully deposited on the glass before starting the flow. A silicone oil (Dow Corning 200, Midland, MI, USA) was selected with different viscosities (20, 100, and 200 cSt) depending on the Reynolds number investigated. This kind of oil was selected based on its relative insensitivity to temperature and ease of use. The images of the droplets were acquired using a Nikon (Minato, Japan) D5800 reflex camera with an observing angle of 45° to maximize the reflections from the oil film. The illumination was provided by a sodium lamp with a characteristic wavelength of 589 nm, positioned at 90° to the optical axis of the camera. A reference image of a calibration target in the measurement plane was used to calculate a de-warping function to correct the perspective distortion and to obtain the calibration factor to convert pixels into physical units (mm).

The conventional processing of droplet images to obtain a WSS measurement involves several steps. First, a manual selection of the IW of each experiment is required. Selecting an appropriate IW is crucial to minimizing errors. The area must be free from artifacts like edge effects, non-uniformities in lighting, or dust particles, ensuring a clear visualization of the interference fringes. Specifically, for each frame, an IW of 3 × 201 pixels with a uniform fringe distribution in the middle of the droplet was manually identified. The data are converted into a 1D line signal representing the brightness variations in the fringes. The distance between consecutive fringes Δx is determined for each frame from the distance between consecutive signal peaks. Finally, the value of WSS is computed from a linear regression of the evolution of Δx as a function of time, following Equation (4).

### 3.3. AI-Assisted OFI Measurements

**Figure 3:**
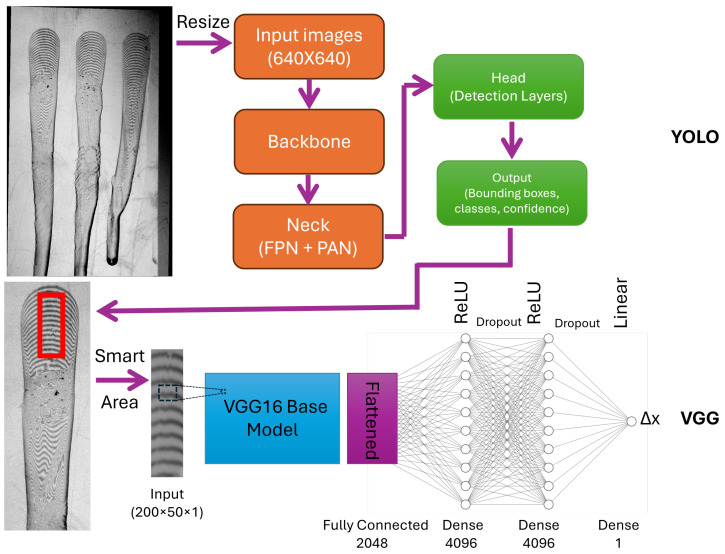
Schematic of implemented combined CNN algorithms. The presented architecture includes three parts: first, YOLO-v8 for the droplet detection task, second, smart interrogation window detection, and third, modified VGG16 for Δx prediction.

The proposed AI-assisted OFI (AI-OFI for short) uses a combination of two CNN algorithms to achieve automated measurement of WSS: a YOLO-based network [40] for the detection of one or more droplets and a VGG16-based network [41] for the prediction of Δx. Additionally, an extra step was introduced after the droplet detection to identify the optimal IW to be used by the VGG16 network. A schematic of the overall algorithm is presented in Figure 3.

#### 3.3.1. YOLO Algorithm

Normally, experiments are conducted with more than one droplet in a single frame, so detecting each droplet is the first task. AI-OFI uses the object detection YOLO-v8 (version 8) algorithm to find and crop each droplet in real-time and automatically. Figure 3 shows different internal steps of YOLO-v8, which receives input 640 × 640 images of three droplets and gives out each droplet’s image separately. Resizing of the input images is automatically performed by YOLO, so AI-OFI does not need any specific input image size.

#### 3.3.2. VGG16 Algorithm

The next step is identifying the Δx of each droplet, which we decided to use VGG to handle. In our case, VGG16 showed the best results. The basic version of VGG16 is designed for classification tasks, but our problem is regression. In this regard, we added a flattened layer and two dense layers with ReLU activators, each followed by dropout layers and a dense output layer with a linear activator. The mentioned modified VGG16 is illustrated in Figure 3, VGG. In the traditional method, as explained in Section 3.2, the results are very sensitive to the choice of the IW, which must be carefully selected manually. The VGG16 network uses as input a larger area of 50 × 200 pixels and is less sensitive to variations or distortions of the fringe patterns. On the other hand, a correct localization of the IW inside the cropped droplet image is required for optimal results.

### 3.4. Smart Interrogation Window Detection

**Figure 4:**
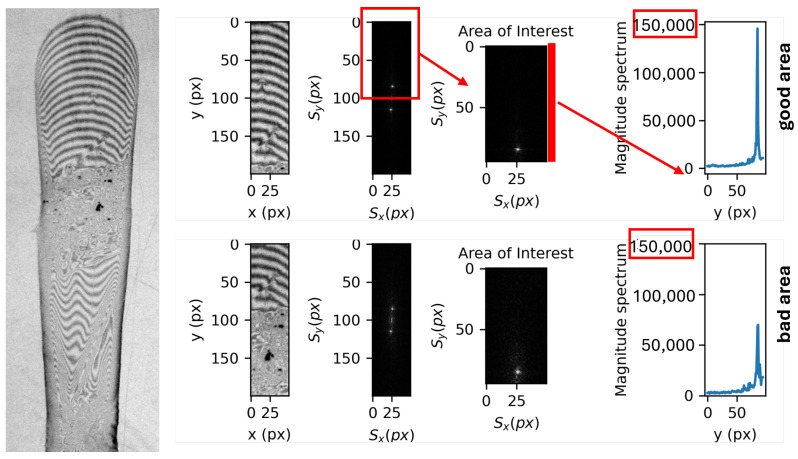
Smart interrogation window detection by applying 2D Discrete Fourier Transform for good and bad interrogation windows. Distances are given in pixel units (px).

Automatically defining the best possible IW for each droplet was the next challenge. Due to different physical or operational problems, in a large number of experiments, we have droplets with some ruined areas where the VGG16 algorithm would fail. In most cases, however, the same droplets also have acceptable areas that can be used in this method. To correctly identify the optimal position of the IW, the idea is to use a method to detect the area with the most repeating patterns. In this regard, we have a sliding 50 × 200 pixel window that moves in the center, covering all relevant areas of the droplet image. As can be seen in Figure 4, we might be faced with a good (yellow window) and a bad (red window). To detect and quantify differences between the two frames, we applied the 2D Discrete Fourier Transform (DFT) [42]

(5) $$F\left(S_{x},S_{y}\right)=\sum_{x=0}^{M-1}\sum_{y=0}^{N-1}I\left(x,y\right)\cdot e^{-2\pi i\left(\frac{S_{x}x}{M}+\frac{S_{y}y}{N}\right)}$$

where I(x,y) is the image intensity as a function of the discrete coordinates *x* and *y*, in pixels, and $\left(S_{x},S_{y}\right)$ are the coordinates in the transform space, corresponding to spatial frequencies. The third column of images in Figure 4 shows the magnitude spectrum of the complex-valued Fourier transform $F(S_{x},S_{y})$ for two exemplary IWs. Besides a peak at the origin, which is disregarded, a second major peak is expected in correspondence to the spatial frequency of the fringes. The magnitude of the peak is proportional to the consistency of the fringe pattern and is used to identify the optimal IW. The calculation is implemented using a 2D Fast Fourier Transform (FFT), which is computationally very efficient and has a negligible impact on the overall processing time.

## 4. Results and Discussion

The development and testing of the proposed AI-OFI approach were based on three datasets, corresponding to three measurement campaigns. The structure of each dataset, including the number of individual experiments (cases) and the number of frames per experiment, is summarized in Table 1. The datasets *Train* and *Test2* were obtained from measurements in the CICLoPE long pipe using the setup described in Section 3.1. In particular, the dataset *Train* was used for training the CNNs. *Test1* was obtained in a zero-pressure-gradient turbulent boundary layer facility at the Faculty of Aerospace Engineering in Delft University of Technology; further details of the campaign are given in [43]. The ground truth (Δx) for *Train* and *Test1* was derived from manual OFI measurements, following the procedure in Section 3.2. The ground truth ($\tau_{w}$) for *Test2* was instead calculated from the measurement of pressure drop across the CICLoPE long pipe.

The first step was to train the VGG16 for the regression tasks of Δx determination, using the *Train* dataset. The input images were gray-scale images with dimensions of 50 × 200 pixels, corresponding to an IW containing a part of an individual droplet (see Figure 3). The training dataset included 18 experiments, each of which consisted of 80 consecutive frames, and each frame had up to 3 droplets, corresponding to a total of 4160 different individual pictures of single droplets. By considering that the average size of each droplet was around 200 by 700 pixels, by sliding the IW randomly 10 times, it was possible to collect a total 41,600 labeled images. 80% of the training data was directed to the training process, and 20% percent was used for validation and early-stage testing of trained weights. Images are labeled by the values from the traditional OFI method. As we mentioned before, generalizing our training setup is important for predicting unseen data, and in this regard, we applied data augmentation methods such as artificial lighting and noising. Unseen cases refer to data acquired under experimental conditions that differ from those used for training, such as variations in imaging setup, acquisition parameters, or operator handling. These cases are not represented in the training dataset and therefore pose a greater challenge for the trained model, serving as an assessment of its generalization capability to previously unseen and potentially more complex scenarios. Data augmentation is applied to improve robustness to illumination and noise variability. Image intensities are randomly scaled within ±40 of their original values and further transformed using a nonlinear intensity adjustment drawn from the same ±40% range to simulate varying lighting conditions. The resulting images are normalized to the [0, 1] range. Additionally, zero-mean Gaussian noise with a standard deviation of 3 intensity units (8-bit scale) is added to emulate sensor noise.

For training the YOLO, we used the images of the same campaign (*Train*), but in this case, we were able to use a pre-trained model of YOLO-v8. It should be mentioned that just the campaign *Test2* has pressure drop data, and we used it for the very final testing of the AI-OFI method.

**Figure 5:**
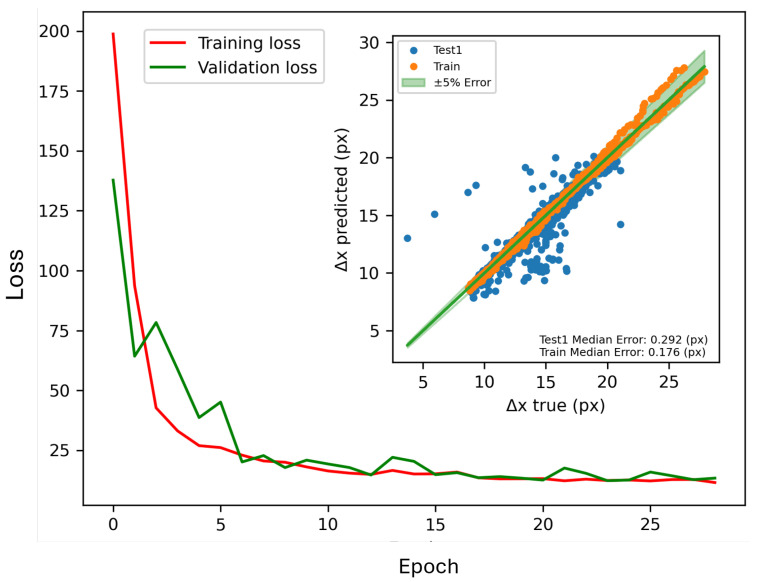
Training loss and validation loss of modified VGG16 training and prediction results for two series of unseen data. Fringes distances (Δx) are presented in pixel units (px).

After training, we presented the modified VGG16 with 44,085,953 trainable parameters for 28 epochs with early stopping, and we achieved the illustrated results in Figure 5. Results show well-converged training and validating loss of training in which no signs of overfitting can be seen. Moreover, true versus predicted results of the initial testing setup, including data from both *Train* and *Test* campaigns, are plotted, and the presented results show the median error of 0.29 and 0.17 pixels for *Test1* and *Train*, respectively. Despite the relatively low error in the prediction of both campaign data, some outliers belonging to *Test1* are visible in the plot, which are low in number in comparison with the total number of cases. Moreover, these outliers could be the result of inaccurate output from traditional OFI. Deeper studies in more unseen cases show the same results, which will be presented in the *Test1* campaign results.

**Figure 6:**
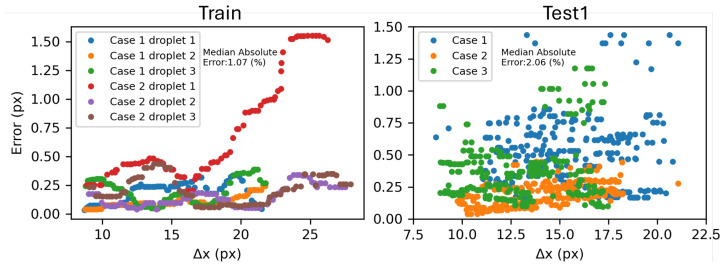
Prediction error compared with Δx for two series of unseen data. Cases 1 and 2 in the left plot correspond to two randomly selected experiments from the training dataset that were excluded from the training process and used only for testing. Cases 1, 2, and 3 represent three randomly selected experiments from the Test1 dataset. Fringe distances (Δx) are presented in pixel units (px).

To have a better vision of predicted results and their dependency on time and fringe distance, predicted Δx in comparison with prediction error is illustrated in Figure 6. Results show that the error value does not change significantly in different Δx values (or during time). The presented results all belong to the experiments in which none of their frames are presented in training (also, in the *Train* campaign, we separated 20% of the experiments for testing/validating). Moreover, the difference between the two plots shows that even unseen cases belonging to the same experimental campaign with training data set (*Train*) are more predictable and familiar to the model, and a real test needs completely new experiments. For instance, in this case, model precision in predicting unseen cases of *Train* and *Test1* was 1.07% and 2.06%, respectively.

**Figure 7:**
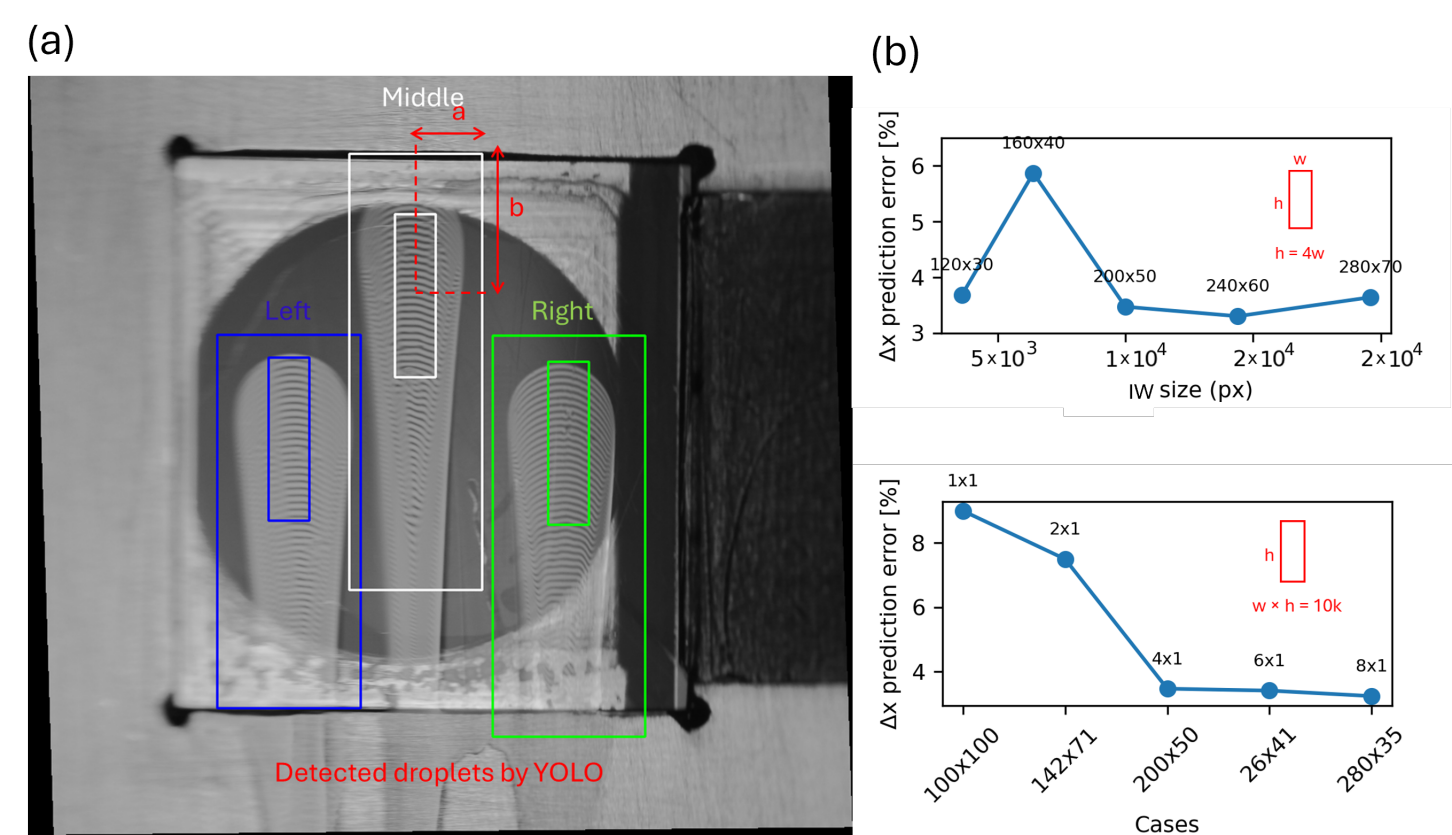
(**a**) The droplet detecting task is performed by YOLO-v8 automatically. (**b**) Interrogation windows size and ratio analysis in Δx prediction by modified VGG16. IW sizes are presented in pixel units (px). A short video of YOLO-v8 and VGG16 task can be found in the Supplementary Material.

In the first major testing stage, we applied our mixed CNN algorithm in 13 unseen experiments of the *Test1* campaign, in which each experiment included 90 frames and three droplets, as shown in Table 1. As can be seen from Figure 7a YOLO is able to detect the interest area of all three droplets successfully. Defining the IW is the next step. The size and ratio of the IW are one of the effective parameters in the prediction and effectiveness of our method. In this regard, we analyzed it in the prediction of VGG16, and the results are presented in Figure 7b. The first plot shows the prediction error of Δx for IW with the fixed ratio of height to width of 4 and different sizes, where the output shows that for sizes more than ($1\times 10^{4}$) pixels, the error is minimal. The second plot illustrates the results of the different ratios and shows significant errors for ratios 1 and 2. Finally, by analyzing the mentioned plots, we ended up with interrogation windows with the size of 50 by 200 pixels.

**Figure 8:**
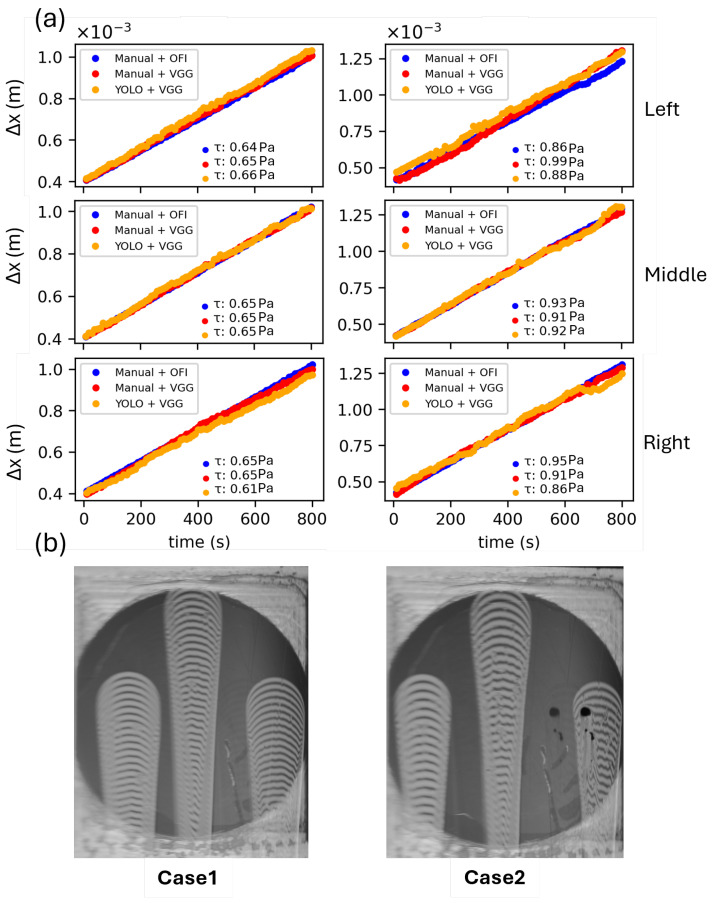
(**a**) Predicted results for the droplets of two unseen cases. In Manual + VGG, the position of the droplet is defined manually, in YOLO + VGG, the position of the droplets is automatically detected by YOLO. For each plot, the corresponding $\tau_{w}$ values calculated with the different methods are reported. (**b**) Snapshot of case1 and case2.

Figure 8 presents the temporal evolution of Δx as measured with three different methods: (1) Blue dots represent data obtained using the OFI method; (2) red dots represent data obtained using the AI method (VGG16) to predict Δx on manually detected droplet images; and (3) yellow dots represent data obtained using the fully automated AI-OFI method in which YOLO detects droplets, smart interrogation windows find the optimal region, and VGG16 predicts Δx. Data are presented for three doplets of two unseen cases belonging to the *Test* campaign.

As can be seen, there is good agreement between different methods. The results show an average error of 0.52 and 7.15% in Manual + VGG with Manual + OFI for case 1 and case 2, respectively. Moreover, the results of YOLO + VGG have an average error of 2.60 and 4.28% with Manual + OFI for three droplets of case 1 and case 2. As can be seen from Figure 8b, the left droplet of case 2 has a ruined area, which raises the concern of the validity of the OFI method. More analysis in the comparison of OFI and AI-OFI, and validation of AI-OFI with ground truth data, has been presented in the following sections.

**Figure 9:**
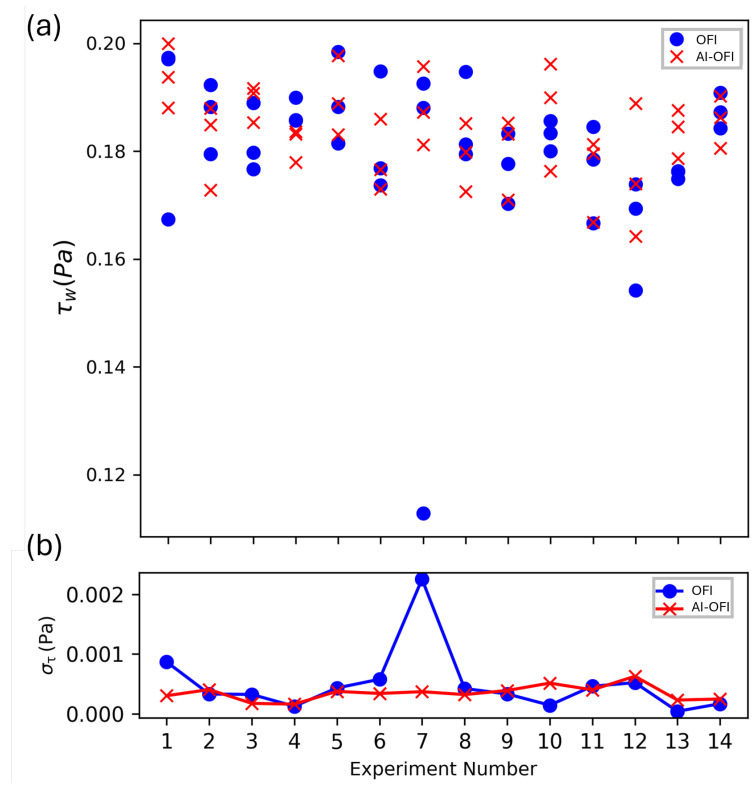
(**a**) Comparison of WSS values for unseen cases of Test1 campaign with OFI (blue circles) and AI methods (red cross). (**b**) Average Standard deviation of tau of each droplet of an experiment with the mean tau value of the experiment. A short video of VGG task can be found in the Supplementary Material.

Moreover, the full autonomous method was applied to all 13 *Test1* cases and resulted in WSS with the OFI and AI methods, and Figure 9 represents the outcomes for different experiments. In principle, all three droplets of each experiment should give out the same τ value due to the same experiment setting; the average standard deviation of the τ value of each droplet of an experiment with an average value of τ of that experiment is illustrated in Figure 9 and shows AI-OFI has less deviation in results in comparison to OFI. Results indicate the new method is able to detect droplets, define the best IW, and obtain the Δx value. Moreover, results show AI has less deviation in predicting τ of each droplet of an experiment and the mean τ of that experiment.

**Figure 10:**
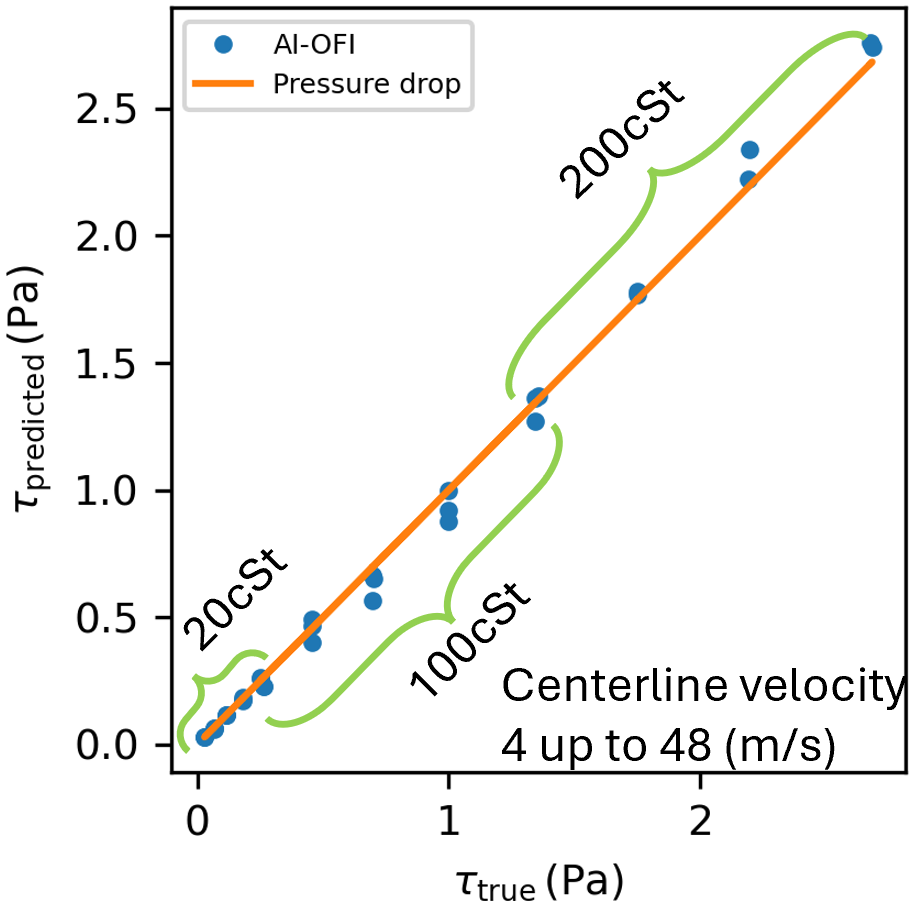
Wall shear stress values for unseen cases of *Test2* campaign with static pressure drop along pipe [44] and AI-OFI methods. Oils with three different viscosities were used in experiments with an air centerline velocity range of 4 to 48 (m/s).

The *Test2* campaign consists of 34 experiments with three different oils (20, 100, and 200 cSt) and a centerline velocity range of 4 to 48 (m/s). This experiment is conducted with two different test section plugs, which leads to different image backgrounds. Since data from the *Test2* campaign are obtained from fully developed turbulent pipe flow, WSS can be directly obtained from static pressure drop measurements through the expression

(6) $$\tau_{w}=-\frac{R}{2}\;\frac{dp}{dx},$$

where *R* is the pipe radius and $\frac{dp}{dx}$ is the pressure drop, evaluated by measuring the static pressure along the last sections of the pipe as described in [1,2,3]. Therefore, these data provide a ground truth that is completely independent of the OFI technique. Comparisons between the fully autonomous AI method and WSS obtained from static pressure measurements in the *Test2* campaign are illustrated in Figure 10. The mean absolute error of this further validation test is 0.032 Pa, corresponding to a ±2.4% deviation of the autonomous AI-OFI method with respect to the ground truth reference.

## 5. Conclusions

This study introduced an automated sensing framework for the measurement of wall shear stress (WSS) based on the Oil-Film Interferometry (OFI) technique, referred to as AI-OFI. The proposed system combines the non-intrusive accuracy of OFI with the data-processing capabilities of modern deep-learning algorithms, enabling a transition from a traditional laboratory visualization tool to a reliable, sensor-like platform for real-time WSS monitoring. The AI-OFI architecture integrates two convolutional neural networks: a pre-trained YOLO-v8 model for automatic droplet detection and a modified VGG16 network for regression-based prediction of the fringe spacing Δx, from which $\tau_{w}$ is derived. In addition, a smart interrogation-window algorithm, based on the two-dimensional Discrete Fourier Transform, autonomously identifies the optimal region for fringe analysis, ensuring robustness against uneven lighting, droplet imperfections, and vibration effects. The method was trained and validated on three independent datasets obtained in two different canonical flow conditions: fully developed turbulent pipe flow in the CICLoPE long-pipe facility, and zero-pressure-gradient turbulent boundary layer, including both manual OFI evaluation and reference pressure-drop data.

Quantitative comparison with conventional OFI and pressure-based WSS estimates demonstrated that the AI-OFI achieves high measurement accuracy, with a mean absolute deviation below 5% across a wide range of flow conditions and oil viscosities. The method also exhibited improved repeatability and reduced sensitivity to operator intervention, effectively eliminating the need for manual selection of interrogation windows. These features confirm the potential of AI-OFI as an autonomous optical shear-stress sensor capable of delivering local, high-resolution, and real-time data.

Future developments will focus on expanding the training dataset to cover a broader range of flow regimes and optical configurations, integrating uncertainty quantification within the deep-learning pipeline, and embedding the AI-OFI system in closed-loop flow-control experiments. The methodology presented here provides a pathway toward next-generation optical shear sensors that combine physical insight with data-driven intelligence, with applications extending to aerodynamics, biomedical flows, and industrial process monitoring.

## Figures and Tables

**Table 1:** Experimental campaigns and datasets used for training and testing AI-OFI.

Dataset Name	Number of Experiments (Cases)	Number of Frames per Experiment ^1^	Centerline Air Velocity Range (m/s)	Oil Viscosity (cSt)	Ground Truth
*Train*	18	80	4–48	20, 100, 200	Manual OFI
*Test1*	13	90	15	100	Manual OFI
*Test2*	34	120	4–48	20, 100, 200	Pressure sensors

^1^ Each frame contains 3 oil droplets.

## Data Availability

The developed code will be made available on the public repository on GitHub. The link will be made available in the published version of the manuscript.

## Supplementary Materials

The following supporting information can be downloaded at: https://www.mdpi.com/article/10.3390/s26020701/s1. Video S1: Full automatic dx calculation.
